# The Impact of Point Mutations in the Human Androgen Receptor: Classification of Mutations on the Basis of Transcriptional Activity

**DOI:** 10.1371/journal.pone.0032514

**Published:** 2012-03-05

**Authors:** Colin W. Hay, Iain J. McEwan

**Affiliations:** School of Medical Sciences, University of Aberdeen, Foresterhill, Aberdeen, United Kingdom; Clermont Université, France

## Abstract

Androgen receptor mediated signaling drives prostate cancer cell growth and survival. Mutations within the receptor occur infrequently in prostate cancer prior to hormonal therapy but become prevalent in incurable androgen independent and metastatic tumors. Despite the determining role played by the androgen receptor in all stages of prostate cancer progression, there is a conspicuous dearth of comparable data on the consequences of mutations. In order to remedy this omission, we have combined an expansive study of forty five mutations which are predominantly associated with high Gleason scores and metastatic tumors, and span the entire length of the receptor, with a literature review of the mutations under investigation. We report the discovery of a novel prevalent class of androgen receptor mutation that possesses loss of function at low levels of androgen yet transforms to a gain of function at physiological levels. Importantly, mutations introducing constitutive gain of function are uncommon, with the majority of mutations leading to either loss of function or no significant change from wild-type activity. Therefore, the widely accepted supposition that androgen receptor mutations in prostate cancer result in gain of function is appealing, but mistaken. In addition, the transcriptional outcome of some mutations is dependent upon the androgen receptor responsive element. We discuss the consequences of these findings and the role of androgen receptor mutations for prostate cancer progression and current treatment options.

## Introduction

Globally, prostate cancer (PCa) is the second most frequently diagnosed cancer of men [1] with the most recent figures from 2010 showing annual deaths of 10,168 in the UK [2] and 32,050 deaths in the US [3] reflecting its prominence as the second leading cause of cancer death in men in Western nations.

Androgen signaling, which is mediated through the androgen receptor (AR), directs development, differentiation and carcinogenesis of the prostate gland [4], [5]. Within prostate tumors, androgen signaling subsequently plays a central role in cancer cell growth and survival [6], [7]. Therefore, androgen ablation through blocking testicular production of androgens, and inhibition of AR function with antagonists constitute the principal systemic treatments for metastatic disease [5], [6], [8]. Although initially efficacious, such therapies fail to provide a lasting cure and the tumor invariably escapes with progression to an exoteric-androgen independent (AI) state [9] which almost invariably leads to death. Hormone refractory tumors continue to express functional AR which plays a critical role in AI cells [10], [11] where it drives a different transcriptome compared to androgen-sensitive cells [12].

The AR, like other members of the steroid hormone receptor family, is a ligand-activated transcription factor which has distinct structural and functional domains [13]: the N-terminal domain (NTD) important for transactivation; the DNA binding domain (DBD) and the C-terminal ligand binding domain (LBD). Upon ligand binding, the AR undergoes conformational transformation facilitating intra- and intermolecular interactions [14]. The transactivational capability of the AR is modulated by several signaling systems [15] through a range of post-translational modifications [13], [16]. Although the AR exerts most of its actions by functioning as a transcription factor binding to specific response elements, non-genomic effects can also contribute to the regulatory outcome. Activation of the phosphatidylinositol 3-kinase (PI3K)/Akt signaling pathway not only regulates AR activity through phosphorylation of the receptor, but also has a major role in the process leading to invasion and metastasis of PCa cells through downstream phosphorylation of affiliated substrates leading to protection from apoptosis and increased cell survival. The AR can stimulate PI3K/Akt signaling by interacting directly with the p85α regulatory subunit of PI3K in response to synthetic and natural androgens [17] through its NTD [18], and by binding and stimulating Akt1 within lipid rafts [19]. Many different processes are involved in the acquisition of hormone resistance [20] and they follow several diverse routes. Activation of sufficient levels of AR in a castration environment can occur through missense mutations within the AR [21], or splice variants, which result in: enhanced binding of androgens; creation of a constitutively active receptor [22]–[25]; promiscuous binding of other ligands [26]–[30] or altered recruitment of co-activators and co-repressors to the NTD and LBD. The levels of AR can be raised through increased expression, altered protein turnover and gene amplification [31]–[33]. In addition, aberrant intratumoral androgen synthesis can lead to activation of AR [34].

Conventional wisdom holds that AR mutations are rare in the early phases of prostate cancer [35], [36] and prevalent in AI and metastatic tumors [37], [38]. In a recent summary of 27 studies, AR mutations in ‘hormone sensitive’ tumors typically ranged from 2 to 25%, while in ‘hormone refractory’ disease the incidence was 10 to 40% [16]. Because the AR gene is located on the X chromosome, its hemizygous state in males means that mutations have a direct phenotypic manifestation. Previous studies on AR mutations have either simply reported the presence of specific mutations in prostate cancer biopsies or analyzed a select few examples using incompatible methodology, thus precluding meaningful comparison of the consequences of the mutations. Given the crucial importance of AR in all stages of prostate cancer progression and the paucity of data on the outcomes of mutations, we have undertaken a comprehensive study of 45 mutations which span the entire length of the protein and are predominantly associated with high Gleason scores and metastatic tumors.

Our analysis of the impact of the point mutations on the receptor's transactivational activity using a cell culture model system revealed several significant findings. We report the discovery of a novel prevalent class of AR mutation that possesses loss of function at low levels of androgen which transforms to a gain of function at physiological levels. Mutations leading to constitutive gain of function are uncommon, whilst the majority of mutations result in either loss of function or no significant change from wild-type (WT) activity. Therefore, the widely held opinion that AR mutations in androgen insensitivity syndrome (AIS) and in prostate cancer result in loss and gain of function respectively is at best an over simplification. Furthermore, the transcriptional outcome of certain mutations is contingent on the AR-responsive element. Together with a literature review of the mutations under investigation, this wide-ranging study aims to bolster research into elucidating the physical basis of the effect of mutations on AR function and understanding the consequences for patient responses to androgen ablation therapies.

## Results and Discussion

### Androgen receptor mutations under study

**Figure 1:**
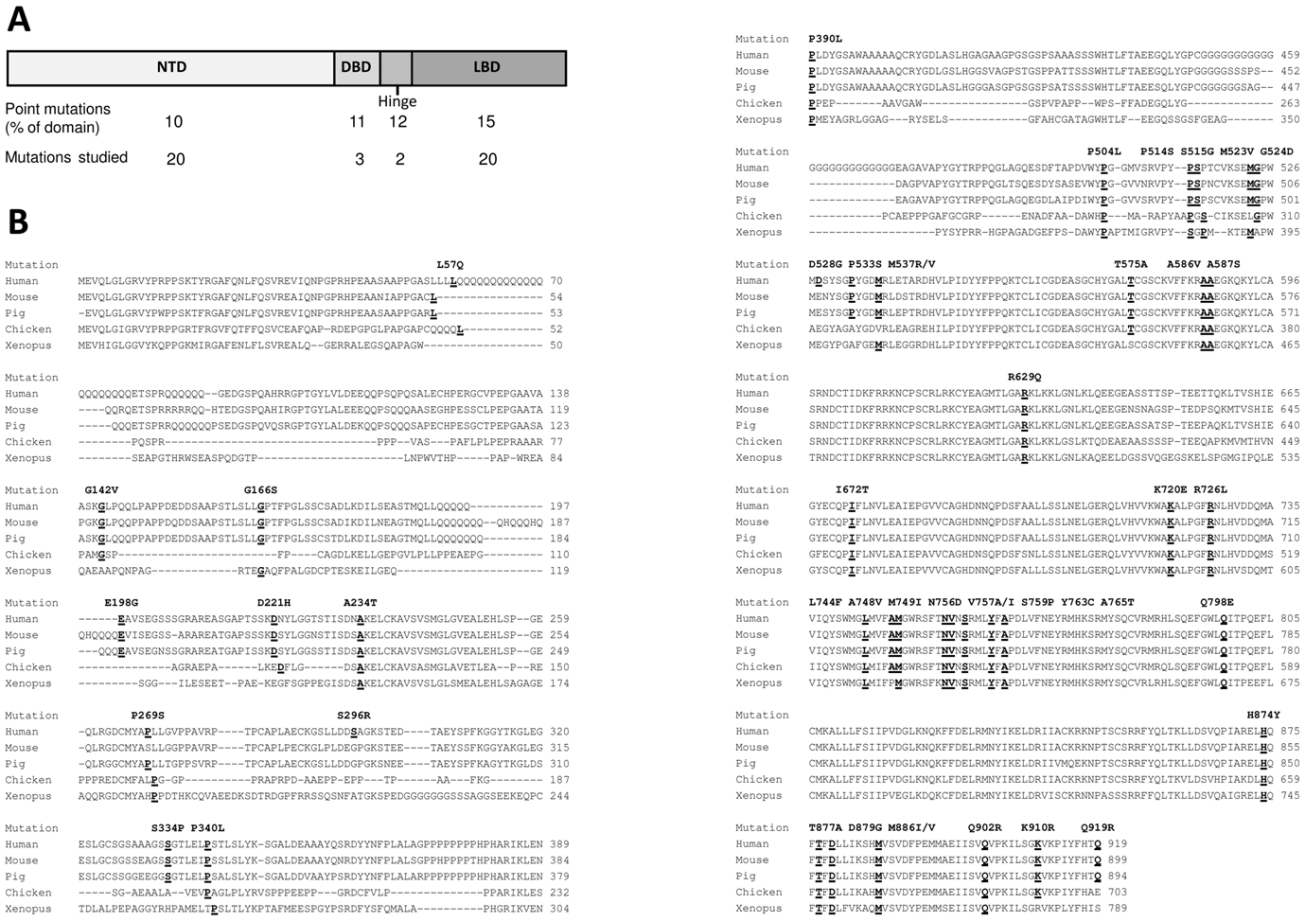
Mutations under investigation. A. Schematic representation of the hAR protein showing the domain-wide distribution of PCa-related point mutations and those forming the basis of the study. B. Androgen receptor homology between human and representative species was determined using ClustalW [96]. The human PCa mutations under study, along with the equivalent residues in other species, are denoted by bold underlined font.

Inspection of the Androgen Receptor Gene Mutations Database (http://androgendb.mcgill.ca) [39] and the literature reveals that prostate cancer-associated single missense mutations occur in the different domains of the AR with relatively comparable frequencies. Within the NTD, DBD, hinge and LBD, 10, 11, 12 and 15% of the residues respectively are currently reported to be mutated (Fig. 1A). The investigation reported here has focused on 45 single missense mutations detected in PCa with metastasis or high Gleason scores, and which extend along the entire length of the protein thereby encompassing all of the different functional domains. The evolutionary conservation of specific residues was also employed as a criterion in the selection of mutations (Fig. 1 and Table 1).

**Table 1:** Type of prostate cancer in which the AR mutations were detected.

Domain	Mutation	Prostate Cancer	Domain	Mutation	Prostate Cancer
NTD	L57Q	Advanced tumor	Hinge	R629Q	AI PCa
	G142V	Gleason 8 AI PCa		I672T	Advanced tumor
	G166S	Locally recurrent Gleason 10 PCa	LBD	K720E	Bone metastases
	E198G	AI bone marrow metastases		R726L	Gleason 7 AI PCa
	D221H	Gleason 9 tumor		L744F	Latent PCa
	A234T	AI tumor in TRAMP mouse model		A748V	Latent PCa
	P269S	Advanced tumor		M749I	Latent PCa
	S296R	AI bone marrow metastases		N756D	AI PCa
	S334P	AI bone marrow metastases		V757A	Metastatic PCa
	P340L	Advanced tumor		V757I	AI PCa
	P390L	AI bone marrow metastases		S759P	Latent PCa
	P504L	Patients receiving combined androgen blockade		Y763C	Latent PCa
	P514S	Gleason 9 AI PCa		A765T	AI PCa
	S515G	Patients receiving combined androgen blockade		Q798E	Advanced tumor
	M523V	Patients receiving combined androgen blockade		H874Y	Metastatic PCa
	G524D	Gleason 7 AI PCa		T877A	Metastatic PCa
	D528G	Advanced tumor		D879G	AI PCa
	P533S	Gleason 9 tumor		M886I	Gleason 7 tumor
	M537R	Patients receiving combined androgen blockade		M886V	Source not known
	M537V	Patients receiving combined androgen blockade		Q902R	Metastatic AI PCa
DBD	T575A	Metastatic PCa		K910R	Gleason 10 tumor
	A586V	Metastatic PCa		Q919R	Source not known
	A587S	Metastatic PCa			

The AR, along with the five other related steroid hormone receptors, originated from successive duplications of an ancestral steroid receptor gene followed by slow sequence divergence in land vertebrates [40]. While this has led to high overall homology between land animals e.g. pig, mouse, chicken and Xenopus AR share 93, 89, 70 and 85% similarity with human respectively [41], functional selection has resulted in the different AR domains exhibiting varying degrees of protein sequence homology between species.

The NTD is by far the least conserved domain with mouse, chicken and Xenopus having only 75, 32 and 34% similarity to human respectively [42]. Alignment of the investigated human AR mutations to the primary sequence of AR in the representative species of mouse, pig, chicken and Xenopus (Fig. 1B) shows that, despite the low sequence identity in the NTD, the vast majority of the studied residues mutated in PCa have been conserved; even when the surrounding motif has not. Only two mutated residues are found in human alone (S296 and D528) and just two are confined to mammals (S334 and P533). Examples of PCa-related residues located within highly conserved motifs include A234, P390, P540, while P269, P340, P514, P515, M523 G524 and M537 which are present in at least four species, though the motif is less conserved in more distantly related chicken or Xenopus. Within the NTD, the region between approximately residues 55 and 230 displays particularly low homology between species, especially in the examples of bird and amphibian shown here. Therefore, in this region we have chosen to examine examples of amino acids implicated in PCa which are present in three or four species as this would suggest a possible role in the mechanics of AR function: namely L57, G142, G166, E198 and D221.

Unsurprisingly, the DBD is virtually unaltered across a wide range of species with 100% homology between the examples shown here; except for two conservative substitutions in Xenopus, one of which T575, has been included in the study. The adjacent hinge region is increasingly viewed as a functionally distinct domain of the AR rather than merely linking the DBD and LBD (see Haelens et al [43]). This region (residues 624 to 676) shows considerably more divergence than its neighbouring domains with mouse, pig, chicken and Xenopus possessing 85, 94, 38 and 59% homology to human respectively. The two highly conserved amino acids R629 and I672 were selected. As the principal function of the LBD is to bind androgen, it is understandably highly conserved with pig, mouse, chicken and Xenopus sharing 98, 100, 92 and 88% homology with human. Accordingly, a series of mutations covering the whole LBD were analyzed.

The sources of the mutations under investigation are shown in Table 1 and the references describing the identification of most are listed in the Androgen Receptor Gene Mutations Database (http://androgendb.mcgill.ca). No mutation was reported to have come from a germ-line source.

### Comparison of reporter plasmids

**Figure 2:**
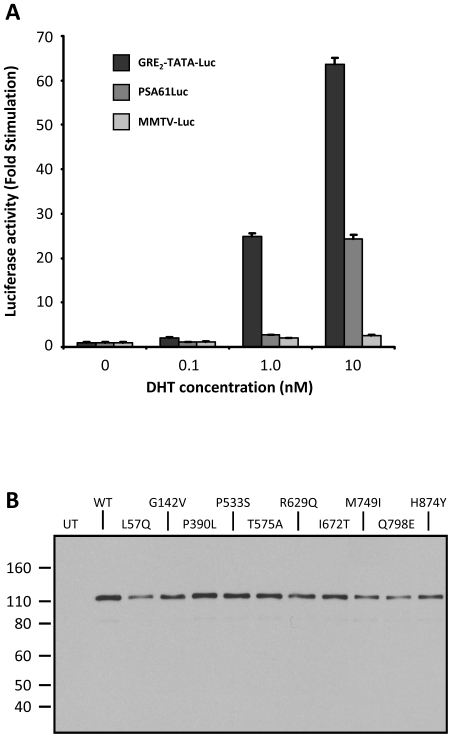
A. Comparison of firefly luciferase reporter plasmids. COS-7 cells were cotransfected with the indicated luciferase reporter plasmid and pSVARo expressing WT hAR. The cells were treated with DHT and firefly luciferase activity was determined. The results were calculated as the fold stimulation of a given reporter plasmid compared to untreated cells transfected with the same plasmid. Values are means of a minimum of three independent experiments performed in quadruplicate ± SEM. B. Western blotting analysis of hAR expressed by mutated pSVARo plasmids. COS-7 cells were transiently transfected with plasmid encoding the indicated mutation, and cell lysates were analyzed for hAR by western blotting. Lysate from untransfected cells (UT) was used as the control.

Initial experiments were performed to determine the most informative firefly luciferase reporter plasmid with which to investigate the functional effects of AR mutations on transactivation activity in the presence of the natural androgen DHT. Comparison of AR transactivation activity on simple or complex promoters present in the plasmids GRE_2_-TATA-Luc, PSA61Luc and MMTV-Luc, co-transfected into COS-7 cells along with a full length human AR expression, revealed that all responded to 10 nM DHT; generating 63.6, 24.3 and 2.6 fold stimulation respectively (Fig. 2A).

The low fold increase in stimulation of MMTV-Luc was due to very high basal luciferase expression in untreated cells which was 5.2 times greater than for GRE_2_-TATA-Luc. This was most likely a consequence of the multiple regulatory elements within the MMTV-LTR and also possibly due to the fact that AR can transactivate the MMTV-LTR without androgen-induced NTD/LBD interaction [44], [45]. Comparable low levels of MMTV-Luc transactivation by androgens have been reported in both non-prostatic cells e.g. COS-1 [46], [47] and PCa cell lines. Within prostate cells, high basal activity and upregulation of between 2 and 9 fold have been observed with DU145 [48], [53], PC-3 [45], [48], [49], CWR-R1 [50] and LNCaP [47]. Oddly, CV-1 African green monkey kidney cells, which are the progenitor of COS cells and the most commonly cited cell line for investigating AR transactivational potentials, routinely exhibit androgen stimulation of MMTV-Luc in the range of 50 to 120 fold [28], [38], [51].

In contrast to MMTV-luciferase, PSA61Luc basal activity was only 0.4 times that of GRE_2_-TATA-Luc and in the presence of 0.1 and 1.0 nM DHT had zero and 1.4 fold upregulation respectively. Plasmid PSA61Luc has been reported to have low responses in the range of 1.4 to approximately 10 fold androgen-stimulation in COS cells [47], [48] and in several PCa cell lines: PC-3 [49], [52]; DU145 [53]; 22RW1 [54]; while LNCaP generally show higher induction of between 40 and 60 fold [55].

Together, COS-7 cells displayed similar characteristics to PCa cell lines and the presence of the large T antigen does not adversely influence AR signaling. Of the three plasmids, only GRE_2_-TATA-Luc responded to low concentrations of DHT with 2.1 and 24.9 fold stimulation at 0.1 and 1.0 nM DHT respectively. As knowledge of the behaviour of the mutant ARs in low androgen concentrations present in castration environments is of major importance, coupled with the robust stimulation at physiological concentrations, GRE_2_-TATA-Luc was selected for comparative analysis of PCa AR mutations.

A panel of expression plasmids was created encoding wild type (WT) AR or each of the 45 PCa-associated mutations by *in situ* mutagenesis. The integrity of the mutations was confirmed by DNA sequencing, and the resulting proteins were validated by western blot analysis; a representative blot is shown in Fig. 2B. The AR mutation expression plasmids were cotransfected with reporter plasmid into COS-7 cells which were confirmed not to express AR (Fig. 2B).

### Effects of mutations on AR signaling

#### Overview

**Figure 3:**
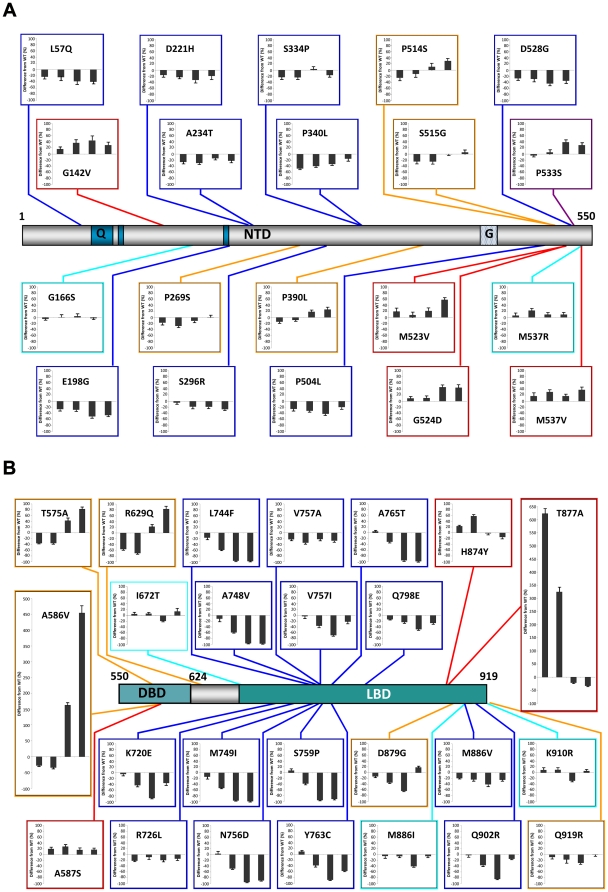
Stimulatory capacity of hAR mutations present in prostate cancer. COS-7 cells were cotransfected with GRE_2_-TATA-Luc reporter plasmid and pSVARo encoding the indicated AR mutation. After treatment with DHT, the luciferase activity was determined. The ability of each AR mutation to upregulate GRE_2_-TATA-Luc was calculated as the fold stimulation at each concentration of DHT compared to untreated cells expressing WT AR. The results are presented as the percentage difference between the mutant and WT AR at each concentration of DHT. Therefore, values below and above zero represent loss and gain of function compared to WT AR respectively. The concentrations of DHT used were: 0; 0.1; 1.0 and 10 nM with the corresponding values displayed from left to right in all charts Values are means of between three and five independent experiments performed in quadruplicate ± SEM. The positions of the mutations are indicated with reference to a schematic representation of the hAR drawn to scale. *Q*, polyglutamine region; *G*, polyglycine region. The different classes of mutation are denoted by colour: *turquoise*, no significant difference from WT; *blue*, loss of function; *plum*, gain of function upon binding of DHT; *orange*, loss of function changing to WT values or gain of function upon binding of DHT; *red*, constitutive gain of function.

Analysis of the impact of the 45 point mutations on the androgen receptor's transactivational activity using our sensitive cell culture model system are summarized in Fig. 3 and reveal several significant findings. Assaying transactivation by AR across a range of DHT concentrations brought to light a predominant novel class of mutations which had loss of function at low levels or in the absence of DHT changing to WT values or a gain of function upon binding of DHT. In addition, the mutations fell into several groups with distinct characteristics. In order to facilitate classification of the mutations, an arbitrary value of 10% difference from WT at two or more hormone concentrations was set as the level for significant dissimilarity. The mutation groups are: 1, no significant difference from WT; 2, loss of function at most or all concentrations of DHT; 3, no significant difference from WT at low levels or in the absence of DHT with a gain of function upon binding of DHT; 4, loss of function at low levels or in the absence of DHT changing to WT values or a gain of function upon binding of DHT; and 5, a constitutive gain of function defined as transactivation activity in the absence of hormone.

Unexpectedly, the majority of mutations (28 out of 45, 62%) led to a loss of function at all concentrations of DHT or were similar to WT; with very few mutations giving rise to constitutive (7 out of 45, 16%) or dramatic gains of function. Significantly, although loss of function mutations were predominantly localized to substitutions in the LBD (14 out of 23 mutations, 61%), the different classes of mutations were distributed along the entire length of the AR protein.

#### NTD mutations

All five classes of mutation were represented within the NTD. Of the five mutations in AR classified as having no change from WT, G166S showed the least variance from the unmutated receptor. The mutation M537R also had minor variation from WT, except at 0.1 nM DHT where 23% gain of function was seen which could have implications in a low androgen environment.

The predominant type of mutation i.e. loss of function, was well represented in the NTD. Mutations L57Q, E198G, D221H, A234T, S296R; S334P, P340L, P504L and D528G all displayed loss of function with E198G showing the greatest reduction (50% at 1 nM) and P340L also being present in AIS. The loss of transactivational ability was generally seen in both basal activity and across a wide range of DHT concentrations. A possible explanation for the loss of function of mutation A234T is that it is located at the start of the highly conserved motif (residues 234 to 247 [56]) immediately carboxyl-terminal of TAU-1 which forms the interaction site for the Hsp70-interacting protein E3 ligase CHIP [57].

Interestingly, there was exiguous rescue at the highest concentration of DHT with D221H, P504L and D528G, while P340L manifested a striking dose-dependent recovery. The S296R mutation has been shown to have altered interaction with the co-repressor N-CoR causing reduced transactivational activity [58]. Mutation of either of the PSTLSL motifs located between residues 159–164 and 340–345 impairs binding of RAP74; the large subunit of TFIIF [59]. Secondary structure simulations for the P340L mutation predict creation of a new α-helix [60], so an obvious possible explanation for its loss of function could be reduced binding with TFIIF. However, there appears to be another dimension to the consequences of this mutation. The co-activator ART-27 binds to the AR NTD and increases transcriptional activity in a dose-dependent manner leading to AR-mediated growth suppression and differentiation [61]. Using a cell-based assay system, ART-27 has been reported to raise the transactivational activity of AR with mutations E198G, P269S and S334P to a similar degree as WT, whereas P340L has an approximately 50% loss of ART-27 stimulation due to increased, but inappropriate ART-27 binding [60]. Given that prostate cancer often has negligible levels of ART-27 expression, and its expression in LNCaP cells inhibits androgen-mediated cellular proliferation, P340L provides a classic example of how a loss of function mutation, also present in AIS, can have the capacity to drive prostate cancer progression through reduced growth suppression.

The novel class of mutation, namely loss of function at low levels or in the absence of DHT recovering to WT values or a gain of function upon binding of DHT was present in the NTD. Mutations P269S and S515G had WT levels of transactivational at 10 nM DHT while P390L (next to the TAU-5 sumoylation site at K386 in the motif IKLE) and P514S acquired 26 and 30% gain of function at 10 nM DHT respectively which would be sufficient to have an impact on AR signaling.

The only mutation to function like WT at low DHT and then gain function compared to WT upon DHT binding was P533S in the NTD. As with other groupings, mutations leading to constitutive transactivation activity were present in all domains of the AR, including four in the NTD. These are G142V, M523V, G524D and M537V and all show constitutive activity in the absence of ligand and modest gain of function at all concentrations of DHT. Mutations M523V and G524D are next to the sumoylation site VKSE (518–522) in TAU-5 and this post-translational modification is considered to repress AR transactivation potential in a promoter-specific manner. As mutation of the sumoylation sites in the NTD increases transactivation [16], it is possible that these prostate cancer mutations may illicit their effect by lessening sumoylation.

#### DBD and hinge mutations

Within the hinge region, mutation I672T has been included in the arbitrary classification of no change from WT due to deviation of less than 10% at 0 and 0.1 nM DHT changing to a 14% gain of function at 10 nM. Interestingly, it showed a loss of function of 19% at 1 nM, which is characteristic of many LBD mutations, suggesting that this region of the hinge adjacent to the LBD allosterically influences ligand binding. The DBD and adjoining sequence in the hinge yielded 3 loss to gain of function mutations and these had amongst the greatest gains seen in the AR. The mutations T575A and R629Q had similar profiles with loss of function at 0 and 0.1 nM DHT changing to 85 and 83% gain of function at 10 nM concentrations respectively. A striking gain of function was seen with A586V with a loss of function at 0 and 0.1 nM DHT being transformed to a substantial 460% increase in transactivational activity at 10 nM. Mutation A587S, which flanks the extremely active mutation A586V in the middle of the α1 linker between the zinc fingers, had constitutive transactivational activity with modest gains of function at all levels of DHT (16 to 28%).

#### LBD mutations

Mutations in the LBD have historically been considered as the most likely candidates for driving PCa, therefore, the finding that the majority of mutations under investigation had no change from WT or loss of function was unexpected. The former were represented by M886I and K910R at the C-terminal end of the LBD and both showed only minor divergence from WT except for distinctive typical losses of function at 1 nM DHT (42 and 27% respectively). Although residue M886 is not essential for ligand binding [51], this mutation has been determined to alter the interaction of the AR with the common co-activators TIF-2 or CBP and the co-repressor N-CoR resulting in elevated and reduced transactivation ability respectively. Taken together, this apparently benign mutation could have significantly altered activity in prostate cancer.

Within the LBD, all but two loss of function mutations were clustered between residues 720 and 798. Of these, half had essentially no transactivational activity at physiological levels of DHT and comprise of L744F, A748V, M749I, N756D, S759P, Y763C and A765T. A similar cluster of loss-of-function mutations was observed in the TRAMP model in castrated mice or animals treated with antiandrogens [62]. It is of note that amino acids in this cluster, which comprise of residues in helix 5 (R752, M745 and M749), a β-strand (F764) and helix 7 (M780 and M787) are directly in contact with hormone. Furthermore, mutation of the corresponding conserved residues in the glucocorticoid receptor revealed an allosteric net work connecting the AF2 surface and the ligand binding pocket [63] suggesting a possible cause for loss of function. The remaining LBD loss of function mutations can be divided into two groups with R726L and V757A showing a modest loss of function at all levels of DHT, and K720E, V757I, Q798E, M886V and Q902R all having a distinctive greater loss of function at 1 nM DHT. This reduction was partially overcome at 10 nM, suggesting reduced affinity for ligand which is ameliorated at higher concentrations. Two of the mutations tested, V757A and Q798E showed impaired binding to the p160 co-regulatory proteins NCoA1 and NCoA2 and impaired N/C-terminal domain interactions (unpublished observations). Six of the LBD loss of function mutations (L744F, S759P, Y763C, A765T, Q798E and M886V) are also present in AIS.

Mutations K720E and R726L, which is implicated in a 6-fold increased risk of prostate cancer [64], reside in a positive cluster in helix 3 with lysine 720 creating a charged clamp with glutamate 897, and both residues participate in the binding of the FxxLF motif present in the AR-NTD as well as the LxxLL found in the p160 co-regulatory proteins [65], [66]. Both mutations impaired binding of NCoA 1, 2 and 3 and disrupted the N/C-terminal interaction as expected (unpublished observations). Residue N756 may be involved in AR dimerization (reviewed in Centenera *et al*. 2008, [14]) and mutation to aspartate resulted in complete loss of function. Similarly, P766 is also critical for dimerization with at least two mutations resulting in AIS and the adjacent prostate cancer mutations studied here, A765T and Y763C, displayed compromised transactivation activity. Residue Q902 in helix 12 forms part of an H-bonding network with Q738, so it is quite possible that positively charged arginine in Q902R disrupts this interaction.

The LBD contained two mutations, D879G and Q919R, which fall within the grouping of loss to gain of function, although recovery to a modest 19% gain of function and WT levels respectively took place at only the highest concentration of DHT. Only two mutations in the LBD, H874Y and T877A, were found to have constitutive activity and both displayed loss of function relative to WT at higher levels of DHT. Threonine 877 participates in hydrogen bonding to the D ring of the steroid ligand and its mutation in T877A brought about by far the greatest constitutive gain of function with a 625% increase in activity compared to WT. The mutation has been studied extensively and also possesses promiscuous ligand binding (see [26]–[30]). As this mutated AR is expressed by the one of the most routinely used prostate cancer cell lines, LNCaP, the undue influence of the constitutive activity on cell-based experiments should be borne in mind.

#### Dependence of hAR mutation effects on the target regulatory element

**Figure 4:**
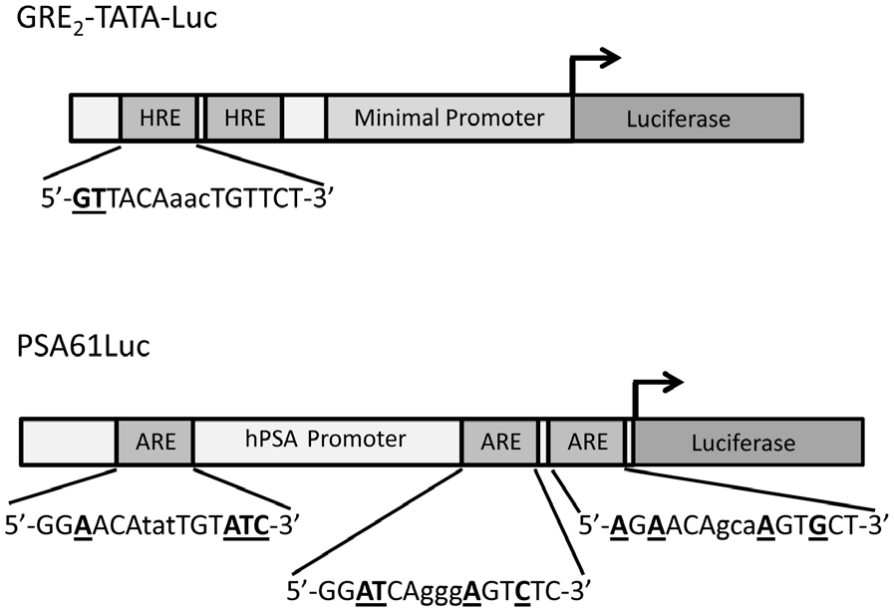
Comparison of the AR-responsive regulatory elements in GRE_2_-TATA-Luc and PSA61Luc. Note that the second copy of the HRE in GRE_2_-TATA-Luc exists as an inverted repeat 29 bp downstream from the first. Nucleotides which differ from the consensus sequences are shown in bold underlined font.

**Figure 5:**
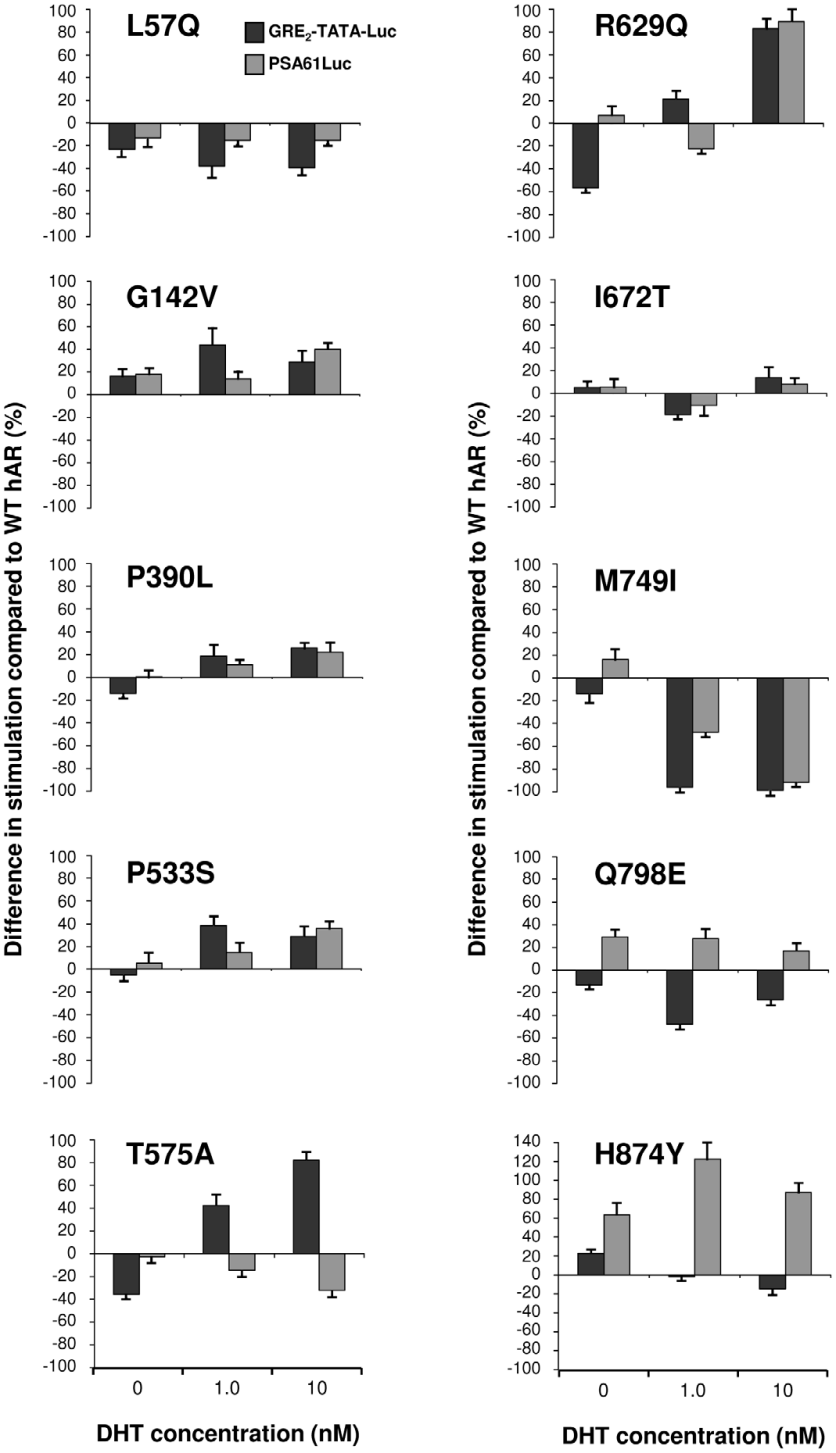
Transactivation activity of mutant ARs on AR-non-specific and AR-specific regulatory elements. COS-7 cells were cotransfected with either GRE_2_-TATA-Luc or PSA61Luc reporter plasmid and pSVARo expressing the indicated hAR mutation. After treatment with DHT, the luciferase activity was determined. The ability of each hAR mutant to upregulate the luciferase reporters was calculated as the fold stimulation at each concentration of DHT compared to untreated cells transfected with the same luciferase reporter and expressing WT AR. The results are presented as the percentage difference between the mutant and WT AR at each concentration of DHT, with values below and above zero representing loss and gain of function compared to WT hAR respectively. Values are means of between three and five independent experiments performed in quadruplicate ± SEM.

To determine whether the transcriptional outcomes of mutations in the AR are influenced by the regulatory element to which the receptor binds, further studies were performed using the PSA61Luc luciferase reporter plasmid. The two copies of the glucocorticoid regulatory element (GRE) present in GRE_2_-TATA-Luc [67], [68] are non-selective for AR whereas the three androgen response elements (AREs) in PSA61Luc are AR-specific (Fig. 4). In addition, the PSA (prostate-specific antigen) promoter driving expression of luciferase in PSA61Luc is a natural DNA sequence of 6.1 kbp which regulates expression of PSA through synergistic cooperative interactions between at least three AREs [48]. Ten representative mutations were assayed for transactivational activity under exactly the same conditions, with the exception that 0.1 nM of steroid was not included, due to the lack of responsiveness of PSA61Luc at this concentration of the androgen (Fig. 2A). The results are shown in Fig. 5 and include the corresponding GRE_2_-TATA-Luc values for direct comparison.

In general, the profiles of PSA61Luc stimulation for the different AR mutations were very similar to those for GRE_2_-TATA-Luc; indicating that the findings in the broad GRE_2_-TATA-Luc study accurately reveal the effects of the mutations. Interestingly, the loss of function in the absence of DHT detected with GRE_2_-TATA-Luc and AR mutations P390L, T575A and R629Q was not seen with PSA61Luc. Instead, there was no significant difference from WT, reflecting probable lower affinity of unactivated AR for the regulatory elements in the PSA promoter as evident in the lower basal activity with WT AR. *In vitro* studies have demonstrated that the isolated AR-DBD (for example [69], [70]) or AR-NTD-DBD [70] exhibited reduced affinity for selective androgen response elements (AREs) relative to hormone response elements, which also bind the glucocorticoid receptor.

The results for the AR NTD mutations investigated with PSA61Luc closely matched those for GRE_2_-TATA-Luc. AR mutation L57Q had loss of function at all concentrations of DHT with both reporters although they were less pronounced with PSA61Luc. Similarly, the profiles of G142V were comparable with both reporters showing constitutive activity and gain of function in the presence of DHT. The difference from WT in upregulation at 1 nM DHT was lower with PSA61Luc than with GRE_2_-TATA-Luc and may again be a consequence of weaker binding between unactivated AR and the androgen-specific AREs in PSA61Luc. Both P390L and P533S, which represented loss to gain of function and WT activity to gain of function respectively, had similar results with the two reporters, albeit with characteristic lower upregulation at 1 nM DHT. Hyytinen *et al* noted that P533S had similar responses to androgens in COS-1 cells using luciferase reporter plasmids driven by either the rat probasin promoter (nucleotides −285 to 32) or two AREs from the first intron of the rat C3 gene in front of a minimal TATA sequence [71].

Mutations within the DBD and hinge domains of the AR would be expected to have the greatest influence on regulating ARE binding and indeed, the profile for T575A in the first zinc finger of the DBD was markedly different for the two reporters with PSA61Luc having a loss of function of 15 and 32% at 1 and 10 nM DHT respectively in contrast to gains of 42 and 82% with GRE_2_-TATA-Luc. Monge *et al* have observed that this AR mutation leads to preferential binding to AR-nonspecific motifs with concomitant increased transactivation and, conversely, to reduced transactivation of AR-specific AREs [72]. Of the two hinge mutations studied, R629Q and I672T, the former exhibited obvious differences in regulatory element activation. Like most of the other mutations exhibiting loss of function with GRE_2_-TATA-Luc in the absence of hormone, there was no difference from WT with PSA61Luc. Also, the results at 1 nM DHT showed a gain and loss of function of 21 and 22% for GRE_2_-TATA-Luc and PSA61Luc respectively. Mutation R629Q resides in the C-terminal extension (CTE) region with the positively charged ^629^RKLKK^633^ motif playing crucial roles in high affinity binding to AREs, nuclear translocation, nuclear sublocalization, intranuclear mobility and transactivation [73]. In addition, post-translational acetylation of the AR at lysines 630, 632 and 633 fine-tunes the action of AR on target promoters and enhancers. Glutamine mimics acetylated lysine through similarity in chemical structure and charge, and mutation of K630 to glutamine produces increased transactivational activity and promotes prostate cancer cell survival and growth [53]. Therefore, the consequences in downstream gene regulation of the mutation of the adjacent R629 to glutamine might arise by interference in acetylation of the ^629^RKLKK^633^ motif or, conversely, by directly imitating acetylation of this influential region. On the basis of homology modeling, I672 resides in a ridge (^670^QPIF^673^) which, along with the carboxy-terminal of helix 9, frames a small highly hydrophobic cleft with the potential to form a protein-protein interaction surface [29]. Mutation to threonine would disrupt the conformation, however, no obvious difference from WT transactivational activity was seen with either reporter plasmid in our studies. This is in contrast to the study cited above which described a nearly three-fold increase with probasin promoter and MMTV-driven reporters in PC-3 and CV-1 cells.

The LBD mutations had a greater dependence on the regulatory elements, emphasizing the importance of interdomain communication for receptor function. While the major losses of function seen with M749I at 10 nM DHT were clearly evident with reductions of 99 and 92% compared to WT with GRE_2_-TATA-Luc and PSA61Luc respectively, there was an important difference in the absence of androgen. With the androgen-specific regulatory element a low level constitutive activity of 16% was observed; against a loss of 14%. It has been proposed that specific loss of function mutations within the LBD including M749I can confer protection from prostate cancer [74], however, this mutation has been indentified in combined androgen blockade relapsed tumors [75] and the constitutive activity may be a contributing factor. The results for Q798E with GRE_2_-TATA-Luc and PSA61Luc were appreciably dissimilar with the modest loss of function seen with the former reporter (up to 40% at 1 nM DHT) being transformed to constitutive gains of function of 29, 28 and 17% at 0, 1 and 10 nM DHT respectively with the latter. Although the values of constitutive transactivation for M749I and Q798E in the absence of DHT are not exceptionally high, the occurrence of constitutively active AR signaling could have profound effects on prostate cancer development over time and explain, at least in these instances, how mutations with apparent loss of function can stimulate prostate cancer progression. Lastly, the PSA61Luc results for H874Y showed increased constitutive activity with a 64% difference from WT, and the small losses of function seen at 1 and 10 nM DHT (2 and 15% differences from WT respectively) became noteworthy gains of function with 123 and 88% increases from WT respectively. Therefore, this mutation located in helix 10/11 with its constitutive activity on both AR-specific and –non-specific regulatory elements, and promiscuous ligand-activation by a wide range of steroid and nonsteroidal ligands [30], [66] represents an especially perfidious prostate cancer mutation.

### Physiological relevance of the mutations

Given that the pathological consequences of AR mutations are strongly influenced by the concentration of androgens within the prostate, a brief overview of current understanding will assist in interpreting the physiological relevance of the data presented in this report. Circulating testosterone is produced principally by the testes with much smaller amounts (about 10%) coming from the adrenal glands. Both testosterone and DHT, which is produced from testosterone by the enzyme steroid 5α-reductase (E.C. 1.3.99.5), bind and stimulate the AR, however, the latter has higher binding affinity and up to 10-fold greater molar potency [76]. Within the prostate gland, DHT is the dominant androgen having an approximately 6-fold higher concentration than testosterone [77]. Steroid 5α-reductase exists as three isoforms: type 1 is present mainly in hair follicles and skin; type 2 is the main variant in the prostate and the recently characterized type 3 isozyme is expressed in AI PCa cells [78]. Conversion of circulating testosterone to DHT is essentially irreversible and intraprostatic levels of DHT are effectively buffered against fluctuations in serum testosterone concentrations by the amplifying action of 5α-reductase [79]. Although serum testosterone levels can decrease in men over 55 to 60 years old, the activity of 5α-reductase isozymes remains sufficiently high that DHT levels are maintained. Studies of serum DHT concentrations in young (19–35 years old) and older men (59–75 years old) receiving testosterone therapy while their endogenous hormone production was suppressed by a gonadotropin-releasing hormone agonist showed no age-related differences. Production of DHT from testosterone displayed saturable Michaelis-Menten kinetics and similar Vmax in both age groups [80].

Intratumoral DHT concentrations can be highly variable and unrelated to circulating testosterone levels due to alternative pathways of production and altered amounts of the 5α-reductase isozymes. PCa cells can convert the blood-borne adrenal steroids androstenediol and DHEA to testosterone [81], [82] which can serve as a substrate for 5α-reductase. Progression to an AI state and metastasis is often accompanied by elevated expression of type 1 5α-reductase over type 2. This not only permits increased conversion of testosterone to DHT, but also allows testosterone to be bypassed with the enzyme catalyzing the 5α-reduction of DHEA-derived androstenedione to 5α-androstanedione, which is then further reduced to DHT [83]. In addition, AI PCa cells can use progesterone as a precursor for DHT [84], and there is growing evidence that they may also have the capacity to carry out *de novo* androgen synthesis [85]. Type 3 5α-reductase expression varies with prostate cell malignancy based on immunostaining. The enzyme is confined to only basal epithelial cells in benign prostate, whereas in high grade PIN it is found in the characteristic neoplastic epithelial cells and in PCa at high levels in most epithelial cells [86].

Several studies on the levels of androgens in prostate biopsies have been carried out and these have been reviewed by van der Sluis *et al* [79]. DHT levels generally ranged between 10.3 and 21.2 nM in patients with benign prostatic hyperplasia and in those with PCa prior to treatment. Importantly, PCa patients undergoing androgen deprivation therapy (ADT) still had intraprostatic DHT levels of between 4.6 and 18.7 nM. Therefore, the third and fourth classes of AR mutation which possess gain of function at higher concentrations of DHT will retain increased transactivational activity in elderly men and even in patients receiving ADT.

Mutations with no apparent change of activity from WT may be able to drive cancer progression though several diverse routes. These include altered binding to co-repressors or co-regulators e.g. M886I, regulatory element-dependant outcome, promiscuous steroid ligand binding, increased half life and raised concentrations due to gene amplification. Mutations with loss of function can be deleterious for the same reasons and also because of diminished action on critical genes downregulated by AR.

### Conclusions

**Figure 6:**
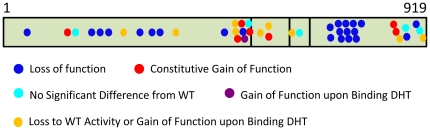
Summary of the distribution of the different classes of mutation along the AR protein.

This analysis examined 45 single missense mutations detected in PCa with metastasis or high Gleason scores, and which extend along the entire length of the protein. Our sensitive assay system uncovered a previously unidentified category of AR mutation that possesses loss of function at low levels of DHT; converting to a gain of function at physiological levels. Importantly, this type of mutation and virtually all of the other classifications were present in all the domains of the receptor (Fig. 6). The preponderance of PCa-associated mutations display loss of function or activity similar to WT and constitutive gain of function is uncommon. Also, there was a lack of an obvious relationship between the type of AR mutation and the severity of the cancer in which it was detected. Four NTD mutations (G142V, M523V, G524D and M537V) have constitutive transactivational activity clearly demonstrating that the NTD has bearing on regulatory element binding; corroborating earlier work showing that intradomain communication between the NTD and the DBD alters affinity for different response elements [70]. It is noteworthy that all of these mutations contain amino acid substitutions capable of forming intramolecular non-covalent bonds, raising the possibility that ensuing conformational changes induce their transactivational properties. The involvement of mutations in all of the other domains of the AR, not just the DBD e.g. T575A, on regulatory element binding is unequivocal. This may be of particular medical relevance as R629Q, M749I and Q798E in the absence of androgen had loss of function with the hormone response element in GRE_2_-TATA-Luc but possessed a constitutive gain of function with the AREs in the PSA promoter. Therefore, mutations categorised as having reduced transactivational activity in one analysis, could well be capable of upregulating prostate cancer-related genes.

The AR is found in distinct cell types in the prostate e.g. luminal epithelial cells and stromal cells, including smooth muscle [5], [7] and evidence from mouse models of cell specific AR knock-out reveals that the receptor can act as a growth promoter and a tumor suppressor, depending on the cell type [87]. AR ablation in epithelial cells leads to increased proliferation and a de-differentiation phenotype [88] while removal of the AR in smooth muscle cells causes increased hormone-dependent proliferation [89]. Interestingly, deletion of the AR in both stromal and epithelial cells reduced proliferation and reduced tumor size in the TRAMP model [90]. Collectively these results suggest that the epithelial AR suppresses the growth signals from stromal cells. Therefore, point mutations in the AR that demonstrate a loss or gain-of function respectively could be important at different stages of disease progression, depending on the cell type and when the mutation occurs.

It is becoming increasingly evident that advanced prostate cancer tumors consist of multiple colonies of cells containing different AR mutations, and therapeutic treatment often results in a culling within the tumor leaving cells with AR mutations conferring a selective advantage to stage a relapse. A fuller understanding of the impact of AR mutations will be of clinical, as well as scientific, value as medical care moves to personalised, targeted treatment based on the manifestation of specific AR mutations in biopsies or circulating cancer cells.

## Materials and Methods

### Cell culture

COS-7 cells [91], an African green monkey kidney cell line, were obtained from The European Collection of Cell Cultures and grown in DMEM containing 5 mM Glutamax and 4.5 g/l glucose (Invitrogen), and supplemented with 10% fetal bovine serum for maintenance or 10% charcoal-stripped fetal bovine serum (both from PAA) for treatment with ligand. Cells were maintained at 37°C without antibiotics in a humidified atmosphere containing 95% air and 5% CO_2_.

### Plasmids and site directed mutagenesis

Three different luciferase reporter plasmids were employed: GRE_2_-TATA-Luc [92], [93]; PSA61Luc [48] and MMTV-Luc [94]. Amino acid substitutions were generated in the full-length human androgen receptor expression plasmid pSVARo [95] using the QuikChange II Mutagenesis kit (Agilent Technologies) according to the manufacturer's methods. The creation of K720E, H874Y, T877A and D879G has been described previously [30] and the other mutations were made with the oligonucleotides and their complements shown in Table S1. The mutations and integrity of final products were confirmed by DNA sequencing.

### Luciferase reporter gene assays

COS-7 cells were seeded in 24-well plates at a density of 1.25×10^4^ cells/cm^2^ and cultured in complete medium containing 10% charcoal-stripped fetal bovine serum for 24 h. Subsequently, they were cotransfected with firefly luciferase reporter plasmid and pSVARo human androgen receptor expression plasmid (150 ng and 75 ng/well respectively) using jetPEI polyethylenimine transfection reagent (Polyplus Transfection) according to the manufacturer's protocol. After 24 h, the medium was replaced with fresh charcoal-stripped medium containing either DHT (Sigma) or solvent vehicle. Twenty four hours later, the medium was aspirated and the wells washed once with PBS. One hundred and ten micro litres Passive Lysis Buffer (Promega) were added to each well and cell lysis was carried out for 15 min with shaking. Cell debris was removed by centrifugation at 13,000 g for 5 min and lysate supernatants were used immediately or stored at −80°C.

Luciferase activity was measured in duplicate by adding 10 µl aliquots of lysate to 96-well plates and using a GloMax 96 Microplate luminometer (Promega) with injection of 100 µl/well in-house luciferase assay buffer containing 13 mM MgSO_4_7H_2_O, 30 mM GlyGly pH 7.8, 1.7 mM Na_2_ATP and 11 µM luciferin (Invitrogen). The luciferase activities, determined as relative light units, were normalized for the amounts of expressed androgen receptor.

### Dot blotting

Androgen receptor levels were determined immulogically by dot blot analysis. Thirty micro litre aliquots of lysate were applied to Hybond ECL nitrocellulose membrane (GE Health Care Systems) using a Hybri-Dot vacuum manifold (BRL). After blocking for 1 h at room temperature in a buffer containing 25 mM Tris–HCl (pH 7.4), 0.14 M NaCl, 2.7 mM KCl, 0.05% (vol/vol) Tween 20 and 5% (wt/vol) nonfat milk powder, the membrane was incubated overnight at 4°C in fresh buffer containing 0.2% (wt/vol) nonfat milk powder and AR441 antibody (sc-7305, SantaCruz Biotechnology) at a dilution of 1∶9,000. The antigen–antibody complex was detected by incubating the membrane for 1 h at room temperature in buffer containing 0.2% (wt/vol) nonfat milk powder and a 1∶5,000 dilution of horseradish peroxidase-conjugated anti-mouse IgG secondary antibody (Sigma) and visualized with an in-house blotting substrate containing 100 mM Tris-HCl pH 7.9, 2.8 mM H_2_O_2_, 1.25 mM Luminol (Sigma) and 0.2 mM p-Coumaric acid.

The amounts of expressed AR were normalized for protein levels which were determined in 96-well plates using the Bio-Rad DC Protein Assay (Bio-Rad) with BSA as a standard. Absorption was measured using a Labsystems Mulitskan MS plate reader and protein concentrations were calculated using Genesis software (both Thermo).

### Preparation of cell extracts

COS-7 cells were plated in 6-well plates at a density of 1.25×10^4^ cells/cm^2^, cultured in complete medium containing 10% fetal bovine serum for 24 h and transfected with pSVARo (1 µg/well) using jetPEI. After 24 h, the cells were given fresh medium and grown for a further 24 h. The wells were washed with ice-cold PBS and the cells were collected by scraping into ice-cold PBS followed by centrifugation at 8,000 g for 3 minutes at 4°C. The cell pellets from each well were resuspended in 40 µl ice-cold lysis buffer containing 100 mM KH_2_PO_4_, pH 7.8, 0.2 mM phenylmethylsulfonyl fluoride and 1× protease inhibitor cocktail (Roche). Cells were lysed by three cycles of vortexing and freeze/thaw using liquid nitrogen. Cellular debris was removed by centrifugation at 14,000 g for 2 minutes at 4°C, and the supernatants were stored at −80°C.

### Western blotting

Samples of 30 µg cell extract were fractionated by SDS-PAGE with 10% acrylamide gels and transferred onto Hybond-P PVDF membrane (GE Health Care Systems) by electroblotting. Thereafter, the membranes were treated as described for dot blotting except that the AR441 antibody was used at a dilution of 1∶900.

## Supporting Information

Table S1 Oligonucleotides (coding strand only) used in creating single point mutations in the hAR.
(DOC)
