# Supplementary material for: The Impact of Point Mutations in the Human Androgen Receptor: Classification of Mutations on the Basis of Transcriptional Activity
Source: PLoS One. 2012 Mar 5;7(3):e32514. doi: 10.1371/journal.pone.0032514 (PMC3293822; doi:10.1371/journal.pone.0032514)
Supplement: Table S1 — Oligonucleotides (coding strand only) used in creating single point mutations in the hAR. (DOC) [file pone.0032514.s001.doc]

Table S1. Oligonucleotides used in creating the hAR mutations.

| Mutation | Oligonucleotide |
| --- | --- |
| L57Q | 5’-cgccagtttgctgctccagcagcagcagcag-3’ |
| G142V | 5’-ccgtggccgctagcaaggtgctgccgcagc-3 |
| G166S | 5’-gttgtccctgctgagccccactttcccc-3’ |
| E198G | 5’-gcagcagggagcagtatccgaaggatccagcagcg-3’ |
| D221H | 5’-gcctcgggggcccccacttcctccaagcacaattacttaggg-3’ |
| A234T | 5’-ctgacaacaccaaggagctctgtaaggc-3' |
| P269S | 5’-gtacgcctcacttttgggagttccacctgcagtgcgtcc-3' |
| S296R | 5’-gcaaaggatccctgctagacgacagggcaggcaag-3' |
| S334P | 5’-gctgcagcaggatcccccgggacacttg-3' |
| P340L | 5’-ggacacttgaactgctgtcgaccctgtctctctacaag-3' |
| P390L | 5’-catcaagctggagaaccttctagactacggcagcgcc-3' |
| P504L | 5’-cctgatgtgtggtatcttggcggcatgg-3' |
| P514S | 5’-ggcatggtgtctcgagtgccctattccagtccc-3' |
| S515G | 5’-gcatggtgtctcgagtgccctatcccggtcccac-3' |
| M523V | 5’-gtcaaaagcgaagtgggcccatggatgg-3' |
| G524D | 5’-gtcaaaagcgaaatggacccatggatgg-3' |
| D528G | 5’-cgaaatgggcccatggatgggtagctactcc-3' |
| P533S | 5’-ggatagctactccggatcctacggggacatgcgtttg-3' |
| M537R | 5’-gaccttacggggacaggcgcttggagactgccag-3' |
| M537V | 5’-ggaccttacggggacgtgcgtttggagactg-3' |
| T575A | 5’-gtcactatggagctctcgcatgtggaagctgcaag-3' |
| A586V | 5’-gaagctgcaaggtcttcttcaaacgcgtcgctgaagggaaacag-3' |
| A587S | 5’-caaaagagcctctgaagggaaacagaagtacttgtgcgccagc-3' |
| R629Q | 5’-ggatgactctgggagctcagaagctgaagaaacttgg-3' |
| I672T | 5’-gaatgtcagcccacctttctgaatgtgctggaagccattg-3' |
| K720E | 5’-gtggtcaagtgggccgaagccttgcctggcttc-3’ |
| R726L | 5’-gccttgcctggcttccttaacttacatgtggacgaccagatg-3' |
| L744F | 5’-gctgtcattcaatactcctggatggggttcatggtgtttgc-3' |
| A748V | 5’-ggctcatggtgtttgtcatgggctggcg-3' |
| M749I | 5’-catggtgtttgccataggctggcgatccttc-3' |
| N756D | 5’-Ggcgatccttcaccgatgttaactccaggatgctctac-3’ |
| V757A | 5’-cgatccttcaccaatgccaactcgaggatgctctacttcg-3' |
| V757I | 5’-cgatccttcaccaatatcaactcgaggatgctctacttcg-3' |
| S759P | 5’-cttcaccaatgtcaaccccaggatgctctacttcg-3' |
| Y763C | 5’-cttcaccaatgttaactccaggatgctctgcttcgcccctgatc-3' |
| A765T | 5’-gatgctctacttcaccccagatctggttttcaatgag-3' |
| Q798E | 5’-gagtttggatggctcgagatcaccccccaggaattc-3' |
| M886I | 5’-tgctaatcaagtcacacatagttagcgtggactttccgg-3' |
| M886V | 5’-atcaagtcacacgtggtgagcgtggac-3' |
| Q902R | 5’-gcagagatcatctctgtacgagtgcccaagatcctttc-3' |
| K910R | 5’-caagatcctttctgggagagtcaagcccatctatttc-3' |
| Q919R | 5’-catctatttccacacccggtgaagcattggaaacc-3' |
